# Pulmonary involvement in transthyretin cardiac amyloidosis: a case report

**DOI:** 10.1093/ehjcr/ytae568

**Published:** 2024-11-09

**Authors:** Michiel Kellens, Petra Nijst, Koen Ameloot, Wilfried Mullens, Philippe Bertrand, Levi Jannis, Jo Van Dorpe, Matthias Dupont

**Affiliations:** University of Antwerp, Prinsstraat 13, 2000 Antwerp, Belgium; Ziekenhuis Oost Limburg, University Hasselt, Synapspark 1, 3600 Genk, Belgium; Ziekenhuis Oost Limburg, University Hasselt, Synapspark 1, 3600 Genk, Belgium; Ziekenhuis Oost Limburg, University Hasselt, Synapspark 1, 3600 Genk, Belgium; Ziekenhuis Oost Limburg, University Hasselt, Synapspark 1, 3600 Genk, Belgium; Ziekenhuis Oost Limburg, University Hasselt, Synapspark 1, 3600 Genk, Belgium; Universitair Ziekenhuis Gent, Ghent University, C. Heymanslaan 10, 9000 Gent, Belgium; Ziekenhuis Oost Limburg, University Hasselt, Synapspark 1, 3600 Genk, Belgium

**Keywords:** Case report, Cardiac amyloidosis, Multidisciplinary, Pulmonary involvement, Transthyretin amyloidosis (ATTR)

## Abstract

**Background:**

Amyloidosis is a systemic disorder characterized by the deposition of misfolded proteins in various organs. While cardiac transthyretin amyloidosis (ATTR) is well-recognized, pulmonary involvement is rare and often overlooked in clinical practice.

**Case summary:**

We present a case of severe, and ultimately fatal, cardiac and pulmonary ATTR amyloidosis in a 67-year-old male. The patient’s initial complaints included dyspnoea and exercise intolerance. Echocardiography revealed isolated concentric left ventricular hypertrophy, and subsequent cardiac MRI suggested cardiac amyloidosis. Additional diagnostic steps, including bone scan and endomyocardial tissue biopsy, confirmed the diagnosis of ATTR amyloidosis. Intriguingly, this case also unveiled concurrent pulmonary involvement, characterized by ground-glass opacities, lymphadenopathy, and impaired lung function. Despite treatment with tafamidis, the patient’s condition deteriorated swiftly. He was admitted to the hospital four months after his initial presentation, and ultimately succumbed to therapy-resistant respiratory distress and heart failure. Post-mortem examination revealed extensive cardiac and pulmonary interstitial ATTR amyloidosis, with the lung exhibiting a fibrotic stage of diffuse alveolar damage.

**Discussion:**

This case highlights pulmonary involvement as a potential contributor to the clinical picture of ATTR amyloidosis. It also emphasizes the necessity for a multidisciplinary approach, heightened awareness, and further research to enhance the detection and management of pulmonary involvement in ATTR amyloidosis.

**Learning points**

- Early consideration and recognition of pulmonary involvement in transthyretin cardiac amyloidosis may be crucial for explaining patient’s symptoms and estimating prognosis.
- Integrating a multidisciplinary approach that involves cardiologist, pneumologists, radiologists, pathologists, and ophthalmologists is essential for comprehensive evaluation and management of transthyretin amyloidosis, ensuring timely identification of potentially overlooked extracardiac manifestations.

**Primary specialties involved other than cardiology** Pneumology, radiology, pathology, ophthalmology.

## Introduction

Amyloidosis is a systemic disorder caused by the extracellular accumulation of misfolded proteins in the form of amyloid fibrils. While there are over 35 protein precursors that can contribute to amyloid deposits, most cases of cardiac amyloidosis are attributed to immunoglobulin light chains (AL amyloidosis) or transthyretin (ATTR amyloidosis).^1^

Although still considered underdiagnosed, cardiac amyloidosis is a well-known clinical entity that ultimately leads to a restrictive cardiomyopathy.^2^ It frequently exhibits early signs that should raise suspicion, such as isolated left ventricular hypertrophy (LVH) or a discordance between increased LV wall thickness and low QRS voltage. Other red flags include extracardiac symptoms affecting the nervous system, GI tract, and musculoskeletal system such as carpal tunnel syndrome, lumbar spinal stenosis, or biceps tendon rupture.^3^ Central in the diagnosis of ATTR amyloidosis is cardiac uptake on bone scintigraphy on the one hand and exclusion of a monoclonal protein on the other hand. This calls for a comprehensive multidisciplinary approach involving specialists from various organ-related fields, as well as diagnostic experts such as radiologists and pathologists. In some instances, advanced inter-specialty care, including collaboration with organ transplant teams, becomes imperative.^3^

Pulmonary involvement is relatively common in amyloidosis but rarely dominates the clinical picture. AL amyloidosis is responsible for most cases, especially those that are clinically relevant. However, pulmonary involvement is also possible in ATTR amyloidosis but likely to be underdiagnosed due to non-specific symptoms.^4^

This report highlights a unique, rapidly evolving case of cardiac and pulmonary ATTR amyloidosis. A chronological overview can be found in *Table 1*.

**Table 1:** Chronological overview of main events and findings

Date	Event(s)	Main finding(s)
Nov-22	First outpatient consultation cardiology (4 November 2022)	Mild exercise intolerance and isolated concentric left ventricle hypertrophy
Dec-22	Cardiac MRI	Suggestive of amyloidosis and identification of lung lesions
Jan-23	Chest and PET CT	Analysis of lung lesions
	Whole-body bone scintigraphy	Perugini grade 3
	First outpatient consultation pneumology	NYHA II
Feb-23	Monoclonal protein identification	Negative
	EBUS and lymph node biopsy	Negative
	Positive endomyocardial tissue biopsy	Definite diagnosis of ATTR amyloidosis
Mar-23	Admission Cardiac Care Unit	Acute respiratory distress
	Right heart catheterization	Combined pre- and post-capillary pulmonary hypertension
	Intubation	Multi-organ failure
	Demise (22 March 2023)	

## Timeline

ATTR, transthyretin; ED, erectile dysfunction; GI, gastro-intestinal; CT, computed tomography.

**Figure:**
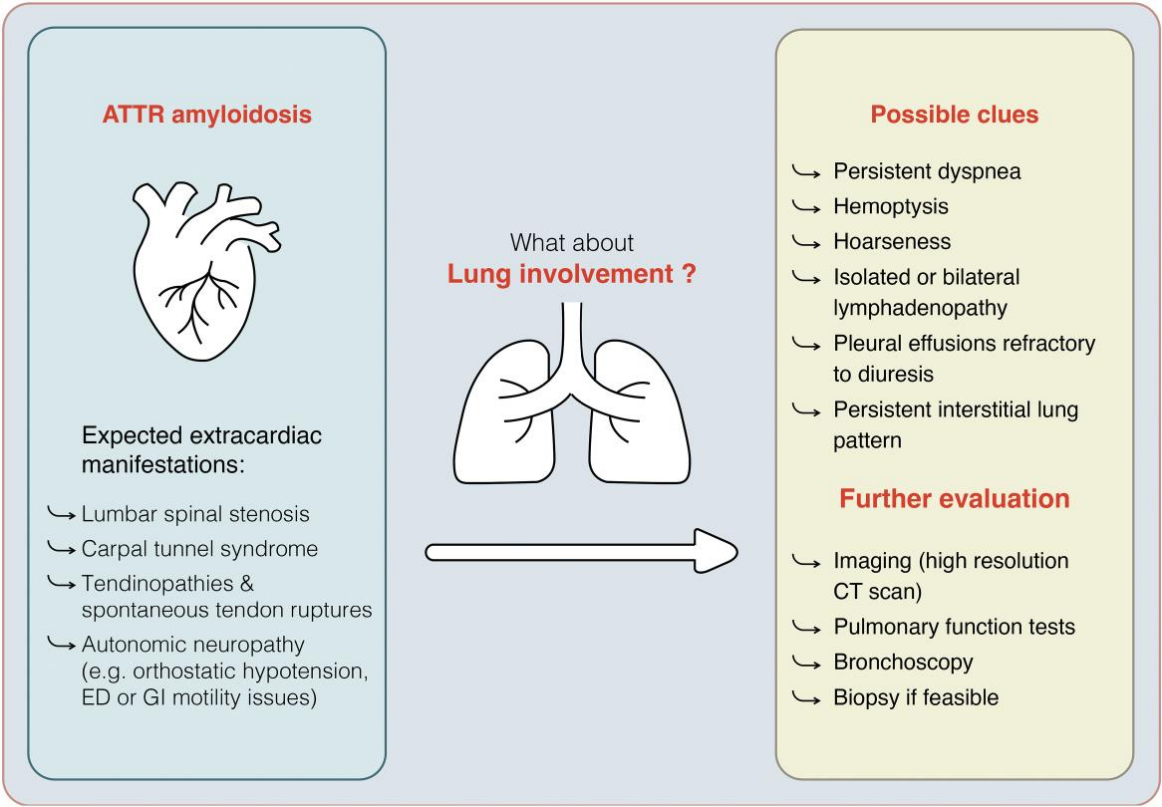


## Case presentation

In November 2022, a 67-year-old man presented to the outpatient cardiology clinic with palpitations and progressive exercise intolerance for several months. The patient had no previous history of heart or lung disease but had undergone surgery for bilateral carpal tunnel syndrome. He was generally active as a runner, cyclist, rock climber, and keen gardener. On physical examination, he had an irregular heart rate of 70 b.p.m. and a blood pressure of 145/86 mmHg. Both cardiac and pulmonary auscultation were normal, and he had no signs of heart failure or volume overload.

**Figure 1:**
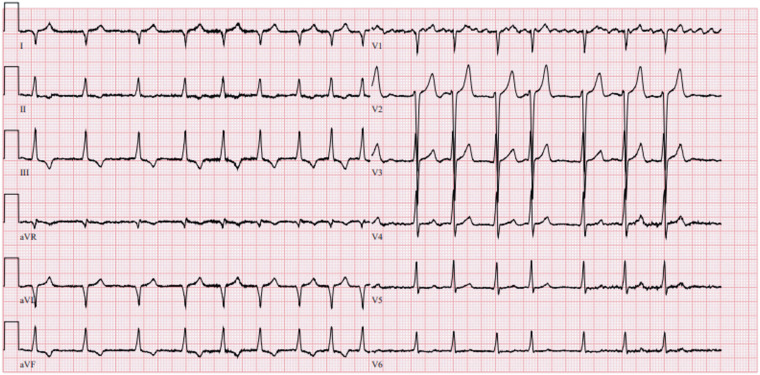
Electrocardiogram at first outpatient contact. Twelve-lead electrocardiogram demonstrates atrial fibrillation with ventricular response of 100 b.p.m., right axis deviation, QRS duration of 98 ms, and T-wave inversion affecting leads II, III, and aVF.

His ECG showed atrial fibrillation, in combination with right axis deviation, and negative T-waves in the inferior leads (*Figure 1*). Initial transthoracic echocardiography revealed a restrictive left ventricle with prominent concentric left ventricular hypertrophy, discrepant with his normal QRS voltage. There was no evidence of significant valvular or other pathology. He underwent successful cardioversion after exclusion of intracardiac thrombi and apixaban 2 × 5 mg and bisoprolol 2.5 mg were started. No other anti-arrhythmic drugs were used. However, because of his unexplained LVH, a cardiac MRI was planned.

**Figure 2:**
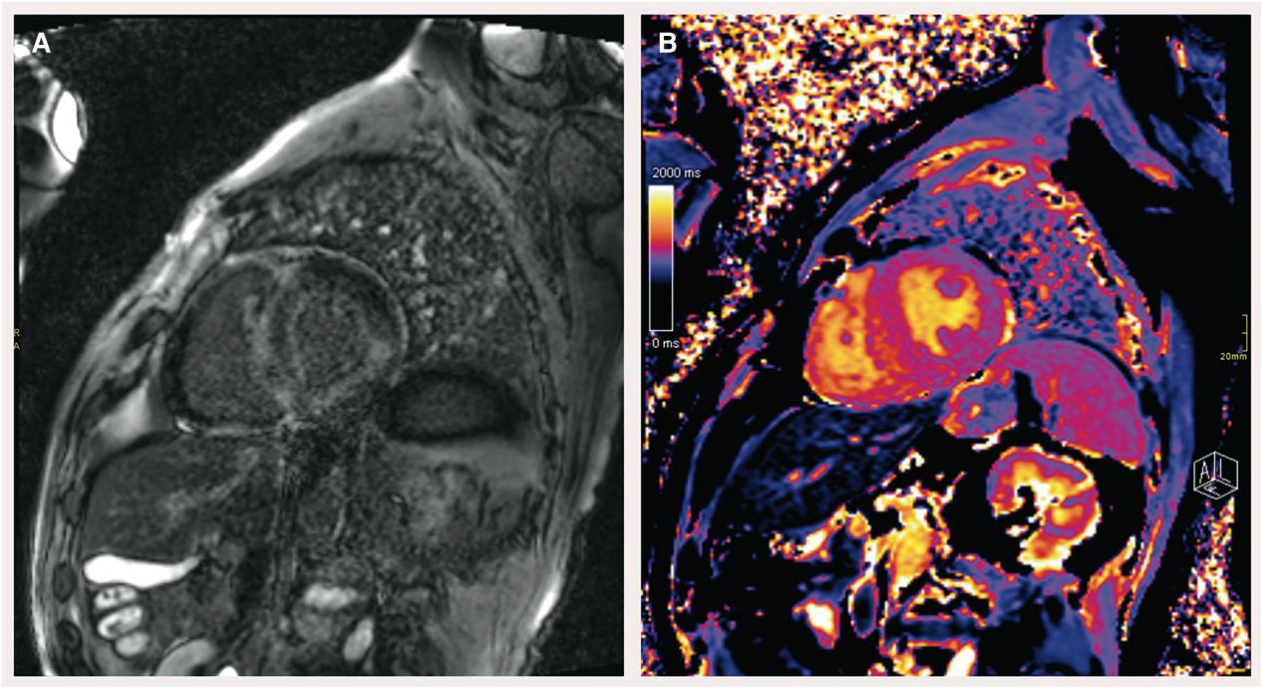
Cardiac magnetic resonance imaging. *(A)* Diffuse late gadolinium enhancement (LGE), pathological contrast uptake in the left ventricular myocardium. Mainly seen subendocardial and subepicardial. Suspected similar contrast uptake in the right ventricular wall. *(B)* Diffusely increased T1 relaxation time in the left ventricular wall, primarily visible in the septal and inferolateral regions at the basal area.

**Figure 3:**
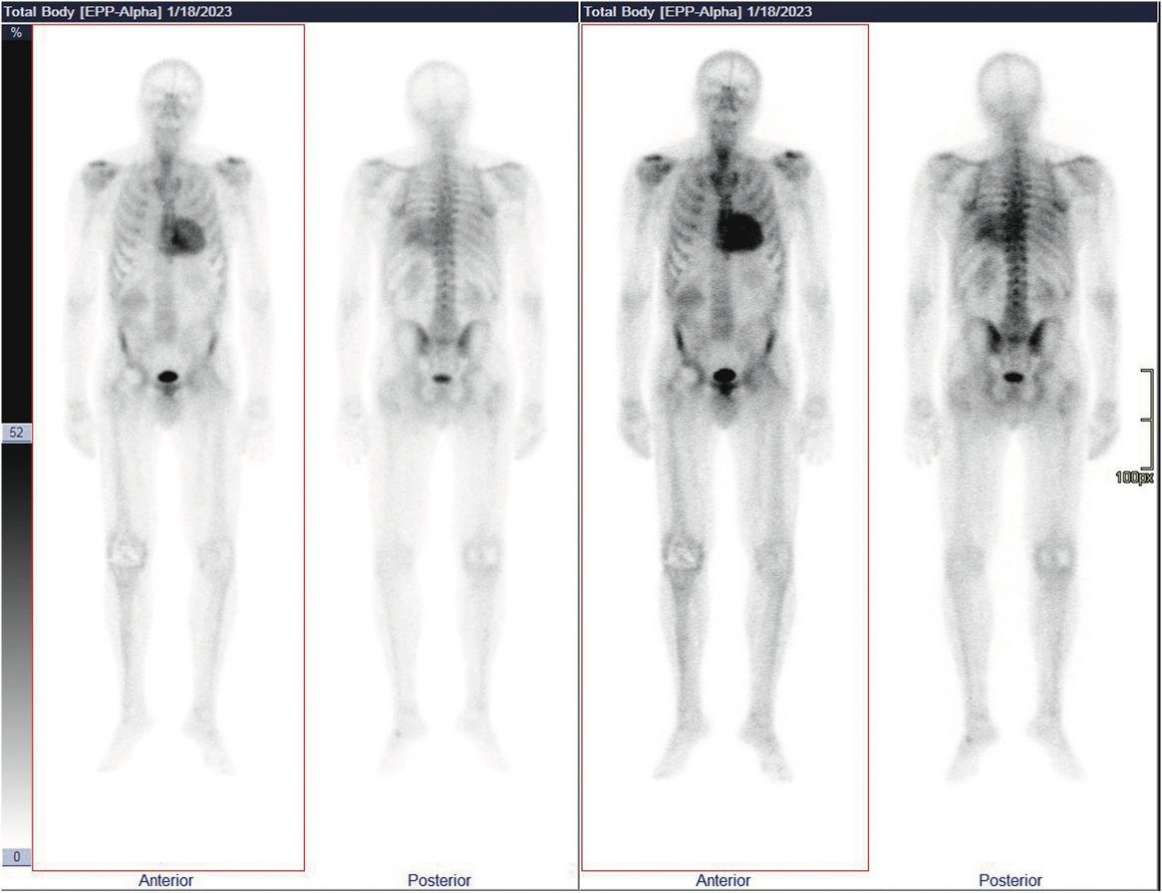
740 MBq Tc-99m-HDP whole-body bone scintigraphy. Whole-body bone scintigraphy showing a highly pathological tracer uptake in the heart, 3 h after i.v. administration of 740 MBq Tc-99m-HDP. Image corresponds to a grade 3 Perugini positive result and is highly suggestive of ATTR amyloidosis.

**Figure 4:**
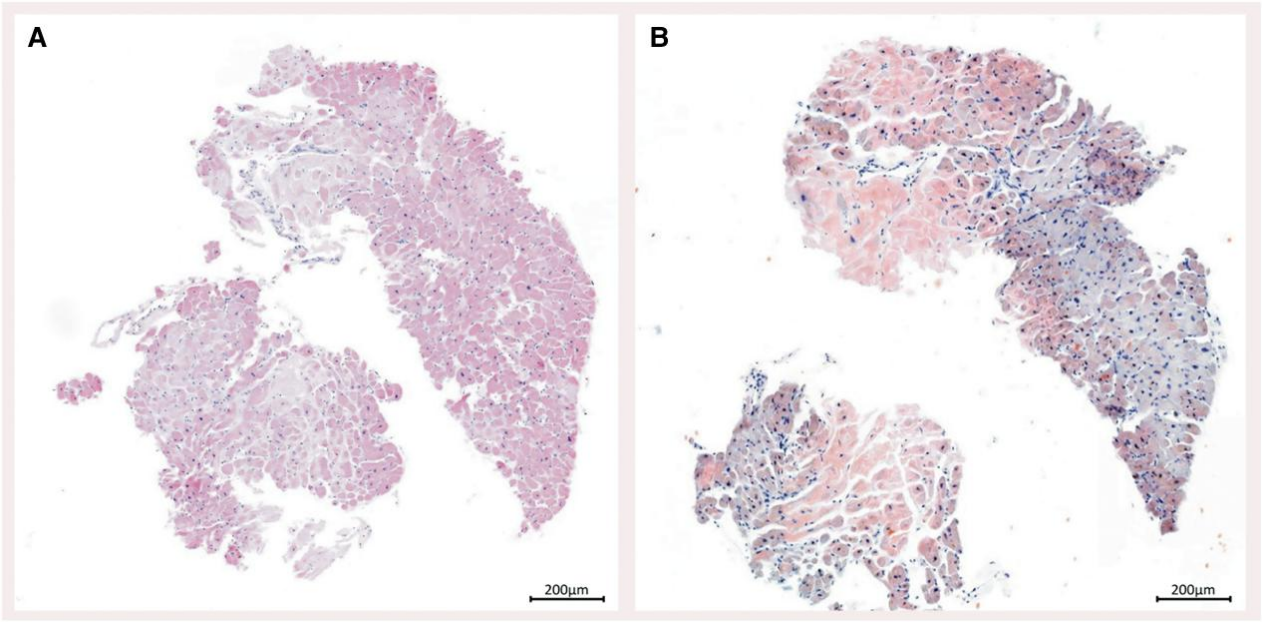
Endomyocardial biopsy. *(A)* Haematoxylin and eosin staining showed amorphous eosinophilic material between the muscle fibres of the cardiomyocytes (×50). *(B)* Upon Congo red staining, these structures exhibit a red colouration, indicative of amyloid presence (×50).

The MRI confirmed a predominantly septal hypertrophic aspect of the left ventricle with an end-diastolic volume of 112 mL and an ejection fraction of 48%. Further imaging showed diffuse prolonged T1 relaxation time and pathological late gadolinium enhancement in the subendocardium and subepicardium of the left and right ventricles (*Figure 2*). In addition, both lung fields showed scattered ground-glass opacities with moderately enlarged mediastinal and hilar lymph nodes. The findings were suggestive of cardiac amyloidosis, but the pulmonary involvement led to a differential diagnosis including sarcoidosis or a separate pulmonary malignancy. For further elaboration, a whole-body bone scintigraphy was performed. Three hours after i.v. administration of 740 MBq Tc-99m-HDP, the scan showed a highly pathological tracer uptake in the heart (Perugini grade 3) (*Figure 3*). Serum and urinary protein electrophoresis and kappa–lambda serum free light chain ratio were normal, ruling out AL amyloidosis. According to the ESC 2021 diagnostic algorithm, the diagnosis of ATTR cardiac amyloidosis could be confirmed.^5^ Due to the rare presentation in combination with the lung involvement, an endomyocardial tissue biopsy was performed. Pathological analysis confirmed Congo red positive amyloid deposition with apple green birefringence under polarized light (*Figure 4*). The combination of these findings confirmed the definitive diagnosis of cardiac ATTR amyloidosis, and tafamidis was started. Later, genetic tests for mutations in the TTR gene were also requested.

**Figure 5:**
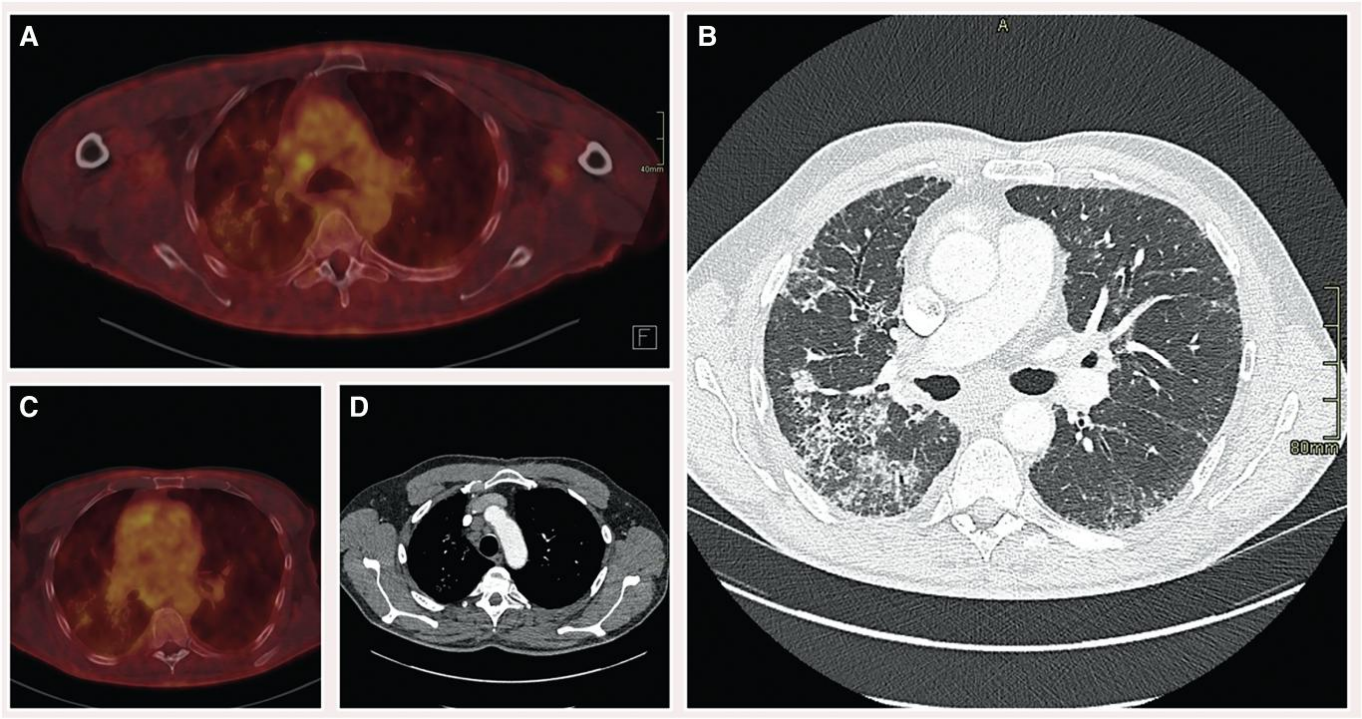
Chest and PET CT. *(A)* PET CT—prominent mediastinal lymph nodes, they appear mildly hypermetabolic. *(B)* CT thorax—scattered reticular to micronodular consolidations with surrounding ground-glass opacities. Predominantly in the right lung. *(C)* PET CT—moderately hypermetabolic lung infiltrates. Right more then left. *(D)* CT thorax—mediastinal and bilateral hilar lymphadenopathy.

Regarding the lung lesions, a thorough multidisciplinary workup was performed. Pulmonary investigation revealed bilateral crepitations and spirometry showed a moderate restrictive pattern with a reduced diffusion capacity of 51%, suggesting interstitial lung disease. Next, chest CT and PET CT were performed. These showed hypermetabolic reticular lung infiltrations with ground-glass opacities (right > left) in combination with mediastinal and hilar lymphadenopathy (*Figure 5*). Endobronchial ultrasound-guided biopsies excluded sarcoidosis and malignancy. The consulting ophthalmologist revealed a highly suggestive image for ocular amyloidosis, including dense vitreous humour on fundoscopy.

One month later, the patient was admitted to the emergency room with haemoptysis and hypoxic respiratory failure. Lab values showed an inflammatory tableau with a C-reactive protein of 254.7 mg/L (nm: <5 mg/L) and a proBNP of 5.949 pg/mL (nm: <125 pg/mL). Haemocultures and extensive bronchoalveolar lavage examinations, including viral and fungus cultures, were negative. His ECG showed a recurrence of atrial fibrillation with a ventricular answer of 123 b.p.m. and known T-wave inversions in the inferior leads. Chest CT excluded pulmonary embolisms but demonstrated an increase in mediastinal lymph nodes and an additional crazy paving pattern spread across both lung fields. His echocardiography demonstrated his known concentric hypertrophic LVH, however now with a small cavity and a very low stroke volume (see Supplementary material online, *Figure S1*). Interventricular septum thickness was 25.3 mm, LV mass 438 g, and LV indexed mass 232 g/m^2^. There were no signs of pericardial effusion.

High-flow oxygen through a nasal cannula was administered, along with intravenous empirical antibiotics and i.v. loop diuretics. Next, amiodarone was started, and a cardioversion was performed to regain sinus rhythm. Both were unsuccessful, and a rate control strategy was adopted. A right heart catheterization (RHC) was performed to guide further treatment. Invasive haemodynamic monitoring showed a combined pre- and post-capillary pulmonary hypertension with high pulmonary capillary wedge pressure and marked elevated pulmonary vascular resistance (*Table 2*). Heart transplantation was considered but was not possible due to multiple organ failure and the age restrictions for transplantation in Belgium. By this time, the X-ray began to show pleural effusions and pulmonary oedema with no improvement of his interstitial and alveolar consolidations. The patient’s respiratory status further deteriorated despite haemodynamically tailored therapy and non-invasive ventilation. The inflammatory markers remained high and proved unresponsive to antibiotic treatment. In the hope of controlling the inflammatory process, a methylprednisolone scheme was started, without clinical success.

**Table 2:** Haemodynamic measurements of right heart catheterization

Haemodynamic parameter	Value
Right atrial pressure	9 mmHg
Right ventricular pressure	
Systolic pressure	72 mmHg
Diastolic pressure	9 mmHg
Mean pressure	30 mmHg
Pulmonary artery pressure	
Systolic pressure	72 mmHg
Diastolic pressure	22 mmHg
Mean pressure	39 mmHg
Pulmonary capillary wedge pressure	22 mmHg
Cardiac output (Fick)	3.4 L/min
Cardiac index (calculated)	1.8 L/min/m^2^
Pulmonary vascular resistance (calculated)	5 Wood units

The patient was eventually intubated due to fulminant acute respiratory distress syndrome. Immediate proning and nitric oxide were applied to improve his ventilation. Despite aggressive supportive therapy, the patient went into multi-organ failure and died 12 days after his admission to the hospital.

**Figure 6:**
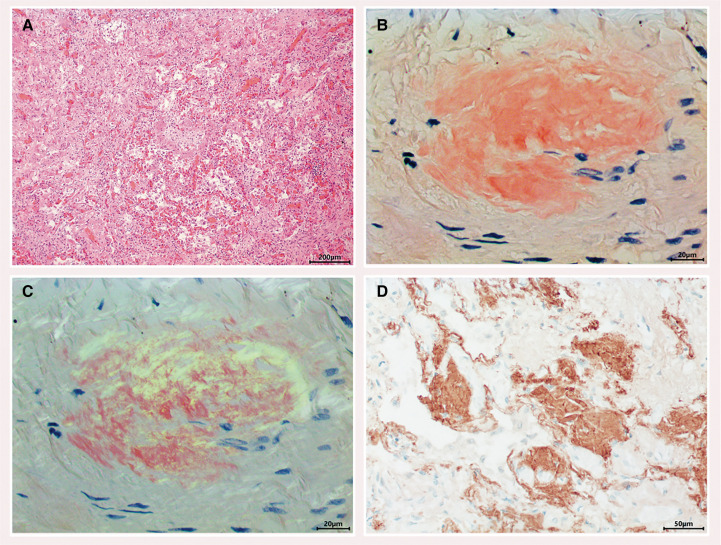
Post-mortem microscopic analysis of lung biopsy. *(A)* Low power view showing severe interstitial lung fibrosis. Some better-preserved alveoli are seen in the middle and lower field of the photomicrograph (haematoxylin and eosin; ×50). *(B)* Congo red staining showing interstitial deposition of amyloid (×400). *(C)* The amyloid is birefringent under polarized light (×400). *(D)* Immunohistochemistry for transthyretin demonstrates impressive deposition of transthyretin amyloid (×200).

After his death, genetic tests for mutations in the TTR gene came back negative. We could conclude that the patient suffered from wild-type ATTR amyloidosis. Genetic counselling was thus unnecessary. An autopsy was also performed. Macroscopic examination revealed cardiomegaly and diffuse consolidation of both lungs. On microscopic examination, extensive cardiac and pulmonary interstitial amyloidosis was confirmed with Congo red staining. There was also amyloid deposition in the media of blood vessels. In addition, the lungs showed important interstitial fibrosis and characteristics of diffuse alveolar damage, mainly hyperplasia of type II pneumocytes (*Figure 6*). Finally, necrosis and other shock patterns were seen in both kidneys and liver.

## Discussion

This case presents a systemic wild-type ATTR amyloidosis with advanced cardiac and pulmonary amyloid deposition in a relatively young patient. An inflammatory stimulus probably aggravated the already existing pulmonary amyloidosis, leading to swift cardiac and respiratory decompensation.

A Mayo Clinic report illustrates how pulmonary amyloidosis is rarely recognized antemortem and underscores its often indolent and asymptomatic nature.^6^ Only one patient (0.01%) died from pulmonary AL amyloidosis, and no cases of clinically significant pulmonary ATTR amyloidosis were reported.^6^ A separate Mayo Clinic review spanning 14 years, reported only one case of antemortem, biopsy-proven pulmonary ATTR amyloidosis.^7^

There are multiple pleuropulmonary manifestations of amyloidosis. Lung involvement can include interstitial or diffuse alveolar-septal amyloidosis, tracheobronchial amyloidosis, pleural involvement, and nodular pulmonary amyloidosis.^8^ Upon retrospective analysis and with knowledge gained from the autopsy, several signs and symptoms in this case now raise some questions. For instance, it’s important to consider that shortness of breath and diminished exercise capacity might too easily be attributed to heart failure, rather than amyloid lung involvement. Therefore, amyloid interstitial lung disease may go unrecognized in cases with concurrent cardiomyopathy. Persistent dyspnoea and interstitial prominence on imaging studies, despite aggressive diuresis, should raise suspicion of amyloid lung involvement. Next, the presence of haemoptysis could have hinted at tracheobronchial involvement. Typically, this is primarily associated with AL amyloid deposition but is also described in ATTR amyloidosis and other forms.^6^

Furthermore, the pleural effusions refractory to diuresis were attributed to cardiac failure but could also be caused by pleural involvement of amyloid deposition. This possibility could not be confirmed during the clinical course, and no pleural biopsies were examined at the time of autopsy. Lastly, because of the extensive lymphadenopathy in this case, nodular pulmonary amyloidosis could also be suspected. The ultrasound-guided transbronchial needle aspiration was unfortunately inconclusive for amyloidosis, so we cannot confirm this suspicion. It must be noted in this specific case, that the patient’s clinical condition at the time of presentation, was already too compromised to allow for invasive diagnostic procedures and limited our options for diagnostic elaboration. Additionally, it must be stressed that these considerations were made in retrospect, with knowledge of the autopsy report, and contain significant hindsight bias. Nevertheless, it’s important to investigate these various clinical presentations, as they may provide valuable insights for recognizing similar cases in the future.

There is no direct clinical implication of diagnosing pulmonary involvement in cardiac amyloidosis since there is no known specific treatment. However, it can explain the excess in symptoms (dyspnoea) and portend a worse prognosis, which is valuable information for both the treating physician and the patient. New emerging treatments targeting ATTR depletion from the body could have large cardiac and pulmonary effects, and influence disease trajectory significantly in these patients.^9^

The median overall survival of ATTR cardiac amyloidosis from diagnosis is 3.6 years.^10^ However, in this case, the patient’s rapid deterioration led to death within one month after definitive diagnosis. If we look at the prognosis based on a staging system by Grogan *et al*., which considers troponin and NT-proBNP, our patient is classified as stage III. This correlates with a respective 4-year overall survival of 18%.^10^ However, when we consider the National Amyloidosis Centre ATTR stage, based on eGFR and NT-proBNP, he is classified as stage II. This correlates with a median survival of 46.7 months.^11^ When we look at prognosis based on haemodynamic parameters, in our case, all conventional parameters appear to be elevated. However, a recent study by Martens *et al*.^12^ interestingly proved that elevated filling pressures are only prognostic at significantly higher threshold values than classic cut-off values. On examining their recommended thresholds, only the mean pulmonary arterial pressure exceeded the prognostic cut-off in our case. Yet, they recognized the cardiac index as the parameter exhibiting the strongest correlation with clinical outcomes and functional status, and in our instance, it was indeed abnormal.^12^ Overall, when we apply different validated staging systems to our patient, we see that they all dramatically overestimate the prognosis of this patient. This can be explained because they are designed solely for cardiac amyloidosis and none of them take pulmonary involvement into account. The disparity between the cardiac prognostic predictions and the trajectory of our case may suggest that pulmonary involvement played a pivotal role in the clinical outcome. The RHC findings reinforce this hypothesis, validating the existence of both pre- and post-capillary pulmonary hypertension and confirming a significant haemodynamic impact of amyloid deposition in the pulmonary arteries.

In conclusion, pulmonary involvement in ATTR amyloidosis is frequently overlooked, even though it is commonly detected during post-mortem examinations. Pulmonary involvement may be the explanation for the excess dyspnoea beyond that explained by heart failure and could impact prognosis significantly. It remains unknown whether molecules targeting depletion of cardiac amyloid might also target and improve pulmonary amyloid.

## Patient’s perspective

Following the patient’s demise, we invited the family for a discussion of the genetic test results. During this conversation, the family exhibited a remarkable understanding of our efforts despite the disease’s rapid progression. They expressed their curiosity about whether this case could enhance current medical knowledge, creating the base for this article.

## Supplementary Material

ytae568_Supplementary_Data

## Data Availability

The data underlying this article will be shared on reasonable request to the corresponding author.

## Lead author biography

Michiel Kellens is currently an internal medicine resident at ZNA Middelheim Hospital in Antwerp. He completed his medical school training at Antwerp University, graduating with great distinction in 2024. His interests within cardiovascular medicine include acute and chronic heart failure management, device therapy, and cardiac intensive care. His current scientific work focuses on incorporating artificial intelligence in echocardiography.

## Notes

**Consent:** The authors confirm that written informed consent was obtained from the patient’s son for submission and publication of this article in line with the COPE guidance.

**Funding**: None to declare.
